# Oncological outcomes of anatomic versus non-anatomic resections for small hepatocellular carcinoma: systematic review and meta-analysis of propensity-score matched studies

**DOI:** 10.1186/s12957-022-02770-4

**Published:** 2022-09-19

**Authors:** Xiao-ming Dai, Zhi-qiang Xiang, Qian Wang, Hua-jian Li, Zhu Zhu

**Affiliations:** 1 Department of Hepatobiliary Surgery, The First Affiliated Hospital, Hengyang Medical School, University of South China, No. 69 ChuanShan Road, Shigu District, Hengyang, 421001 Hunan China; 2 Department of Reproductive Medicine, The First Affiliated Hospital, Hengyang Medical School, University of South China, No. 69 ChuanShan Road, Shigu District, Hengyang, 421001 Hunan China; 3 Department of Education and Training, The First Affiliated Hospital, Hengyang Medical School, University of South China, No. 69 ChuanShan Road, Shigu District, Hengyang, 421001 Hunan China

**Keywords:** Hepatocellular carcinoma, Anatomic resection, Non-anatomic resection, Propensity score matching, Survival

## Abstract

**Background:**

Primary liver cancer is the second-most commonly occurring cancer and has resulted in numerous deaths worldwide. Hepatic resection is of two main types, i.e., anatomic resection (AR) and non-anatomic resection (NAR). The oncological outcomes of hepatocellular carcinoma (HCC) patients after AR and NAR are still considered controversial. Therefore, we aimed to compare the impact of AR and NAR on the oncological outcomes of HCC patients with tumor diameters ≤ 5 cm using the propensity score matching method and research-based evidence.

**Method:**

A systematic literature search was conducted. The main outcomes were disease-free survival (DFS), overall survival (OS), intrahepatic recurrence rate, and extrahepatic metastasis rate. Relative risk (RR) was calculated from forest plots and outcomes using random-effects model (REM).

**Result:**

AR significantly improved DFS at 1, 3. and 5 years after surgery, compared to NAR (RR = 1.09, 95% CI = 1.04–1.15, *P* = 0.0003; RR = 1.16, 95% CI = 1.07–1.27, *P* = 0.0005; RR = 1.29, 95% CI = 1.07–1.55, *P* = 0.008). However, both of the difference in DFS at 7 years and OS at 1 and 3 years after AR versus that after NAR were not statistically significant. Nevertheless, the long-term OS associated with AR (5, 7, and 10 years) was superior to that associated with NAR (RR = 1.12, 95% CI = 1.03–1.21, *P* = 0.01; RR = 1.19, 95% CI = 1.04–1.36, *P* = 0.01; RR = 1.18, 95% CI = 1.05–1.34, *P* = 0.008). The difference in the intrahepatic recurrence rate after AR versus that after NAR was not statistically significant, but the extrahepatic metastasis rate after AR was significantly lower than that observed after NAR (RR = 0.61, 95% CI = 0.40–0.94, *P* = 0.03).

**Conclusion:**

Therefore, AR should be the preferred surgical approach for HCC patients with tumor diameters ≤ 5 cm.

**Trial registration:**

PROSPERO registration number CRD42022330596.

## Introduction

Primary liver cancer is the second-most commonly occurring cancer and has resulted in numerous deaths worldwide [1]. According to the latest GLOBOCAN statistics, approximately 841,000 new cases of liver cancer were reported worldwide in 2018 (4.7% of all tumors), and these resulted in 781,631 patient deaths (8.2% of all tumor deaths). Histological subtypes of liver cancer include hepatocellular carcinoma (HCC), intrahepatic cholangiocarcinoma (ICC), mixed hepatocellular-cholangiocarcinoma, and fibrolamellar carcinoma (FLC). Of these, HCC accounts for approximately 90% of all primary liver cancer cases [2]. Currently, the main treatment modalities for HCC include surgical resection, liver transplantation, transhepatic artery chemoembolization, local ablation, targeted therapy, and immunotherapy [3].

As with other solid tumors, surgical resection is recognized as the treatment of choice for resectable HCC because it is associated with a relatively good prognosis [4, 5]. Hepatic resection is of two main types, i.e., anatomic resection (AR) and non-anatomic resection (NAR). The former is a regular segmental resection process based on the anatomical structure of the liver. The most significant advantage of AR is that it facilitates systematic distribution to the portal vein, which allows for a more complete resection of the lesion with controlled bleeding. In NAR, the extent of resection depends entirely on the tumor distribution, irrespective of the intrahepatic anatomy. In principle, only the complete resection of the tumor (negative margins) is required, thus allowing tumor-free liver tissues to be preserved more effectively [6]. Both AR and NAR are widely used as surgical approaches for the treatment of primary liver cancer.

However, controversies centered on the oncological outcomes of AR and NAR for HCC have been ongoing since several years. Some scholars have argued that AR maximizes tumor resection and improves postoperative survival and thus prefer AR [7–13]. Others argue that there is no difference in postoperative survival after the two procedures [14]. Clinically, AR results in a larger liver section and smaller residual liver volume than NAR. The time required to perform AR is longer, and a tendency of increase in the extent of intraoperative bleeding is observed subsequently. In addition, most patients with liver cancer have different degrees of chronic liver disease and cirrhosis; thus, the incidence of postoperative small liver syndrome or liver failure is higher than that observed with NAR. Because of certain limitations and shortcomings of AR, NAR has become important for the treatment of HCC patients with tumor diameters ≤ 5 cm [15]. In the current case-control study of HCC patients with tumor diameters ≤ 5 cm, it was difficult to obtain a convincing result after comparing the prognosis of the two procedures. The clinical characteristics of the AR and NAR groups, including basic preoperative characteristics, liver function status, tumor number, and vascular invasion were somewhat different, and these variables may affect outcomes. In observational studies, the propensity score matching method (PSM) is frequently used to ensure balanced comparability, to reduce the effects of data bias and confounding variables and to conduct a more reasonable comparison of experimental and control groups [16]. However, no systematic evaluation has been conducted using PSM to date, to address the above-mentioned issues.

Taking these unresolved issues in the clinic into consideration, the current study, which includes an analysis of the most recent retrospective studies, provides evidence-based support that is applicable to clinical practice, [7–14] and uses PSM and meta-analysis methods to systematically evaluate the oncological outcomes of HCC patients with tumor diameters ≤ 5 cm after treatment with AR and NAR.

## Materials and methods

Our work has been conducted in line with Preferred Reporting Items for Systematic Reviews and Meta-Analyses (PRISMA) statement standards and registered in the PROSPERO database (CRD42022330596, https://www.crd.york.ac.uk/PROSPERO/display_record.php?RecordID=330596).

### Inclusion and exclusion criteria

The inclusion criteria were as follows: (1) patients were diagnosed with primary HCC and the study included a comparison between AR and NAR, (2) tumor diameters were ≤ 5 cm, (3) overall survival (OS) and disease-free survival (DFS) after surgery were reported, (4) the findings were reported in the English language, and (5) propensity-score matched studies.

The exclusion criteria were as follows: (1) patients underwent secondary surgery or had metastatic HCC, and HCC patients had tumor diameters >5 cm; (2) valid data such as outcome indicators could not be extracted; (3) animal experiments, reviews, guidelines, meta-analyses, case reports, abstracts, conference proceedings, or discussion of expert opinions; and (4) studies contained repeatedly published information.

### Literature search strategy

The PubMed, Embase, and Cochrane databases were searched until 30 April 2022. The literature search was performed using a combination of subject-related terms and keywords. The search terms included hepatocellular carcinoma, liver cell carcinoma, liver neoplasms, anatomic resection, non-anatomic resection, hepatectomy, and propensity score matching.

### Literature screening and data extraction

Two investigators independently screened the literature and then extracted and cross-checked the data. The literature was screened by first reading the title and abstract, and after excluding irrelevant literature, the full text was read further, to decide whether to include or exclude the study. The extracted data included (1) basic information about the included studies, such as the name of the first author, type of study, and year of publication; (2) baseline characteristics of the study population and interventions; (3) key elements of risk of bias evaluation; and (4) outcome indicators and outcome measurement data of interest.

### Evaluation of literature quality

The risk of bias of included literature was evaluated independently by two investigators and the results were cross-checked using the New Castle-Ottawa scale (NOS), [17] to evaluate the risk of bias. The total score was 9, with a score ≥ 7 being indicative of high-quality literature and a score ≥ 6 required for inclusion.

### Statistical processing

All statistics have been performed using the RevMan 5.3 software. Dichotomous variables were assessed using the relative risk (risk ratio, RR), and their 95% CIs were calculated to determine each effect size. Meta-analysis was performed for all outcomes of interest using the random-effects model (REM). The extent of heterogeneity between studies was determined quantitatively using the *I*^2^ value. Heterogeneity was considered to be present if the *I*^2^ statistic was > 50%. If there was significant heterogeneity (*I*^2^ > 50%) between the results of the studies, the source of heterogeneity was further assessed using sensitivity analysis. Publication bias was assessed with funnel plots. All tests were two-tailed, and differences were considered statistically significant if *P* < 0.05.

## Results

### Literature screening process

**Fig. 1:**
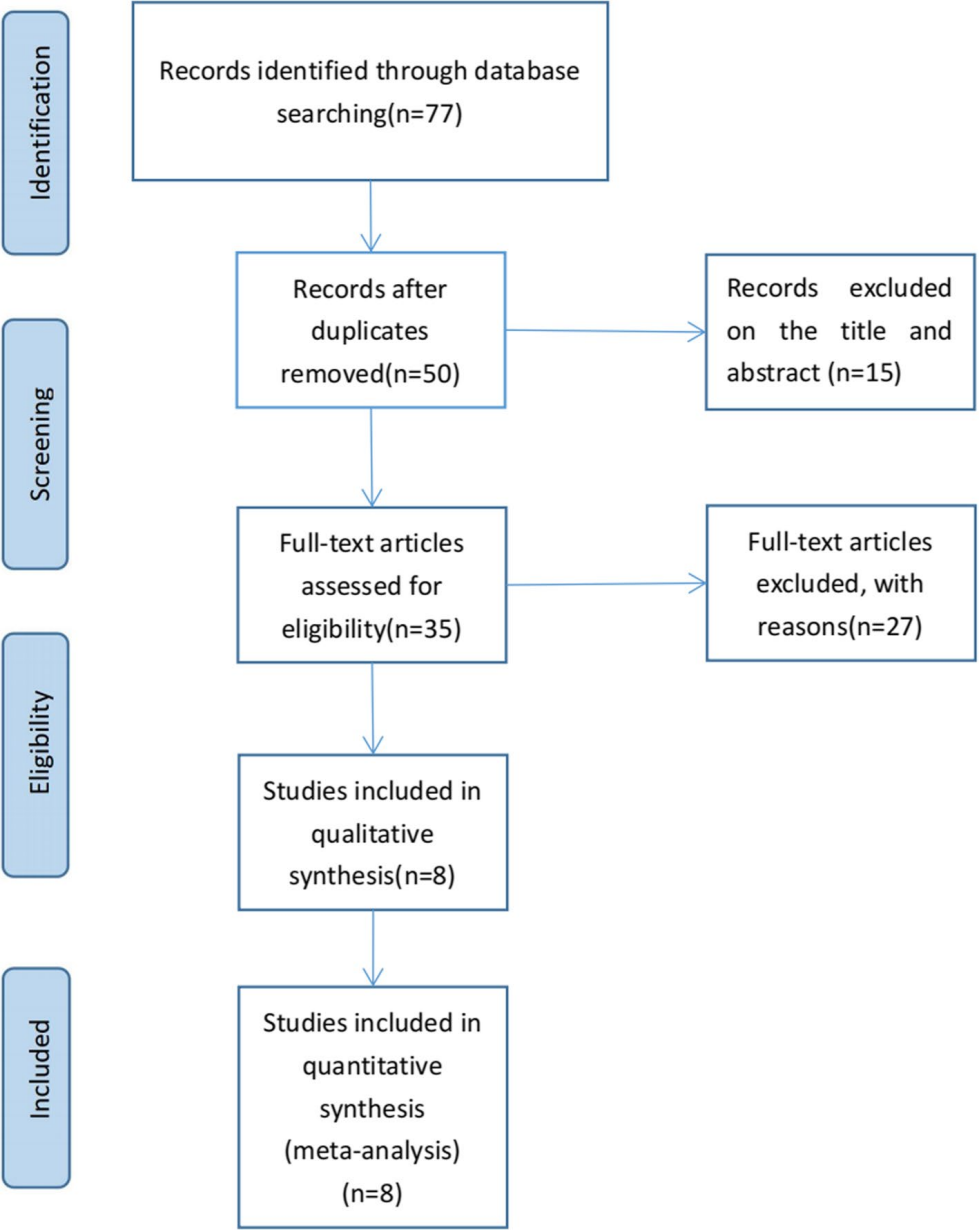
Flow chart of selection process in this meta-analysis

The search strategy and successive steps used during the selection process are shown in Fig. 1. Of the 77 retrieved records, 27 were duplicate studies; 15 were considered irrelevant to the content of the study after reading the title and abstract; the remaining 35 studies were read in their entirety, after which 27 studies that did not meet the inclusion criteria were excluded. Finally, 8 retrospective studies [7–14] with an NOS score ≥ 7 were found to be eligible (Table 1). A total of 1861 patients, including 984 (53%) in the AR group and 877 (47%) in the NAR group, were matched, to assess propensity scores in the literature.

**Table 1:** Details of studies included in the meta-analysis

First author/year	Country	Population Selection	Variables matched	Total Sample	AR	NAR	NOS Score
Cho 2019 [14]	Korea	Small solitary HCC with MVI (< 5 cm)	Age; sex; albumin; AST; ALT; tumor size; vascular invasion; operating time; blood loss	118	59	59	8
Cucchetti 2014 [7]	Italy-China	Child A; early HCC; surgical margin ≥ 1 cm; Single nodule < 5 cm or up to 3 nodules < 3cm	Age; HCV; HbsAg; albumin; MELD score; solitary tumors; size; G3-G4; MVI	298	149	149	7
Haruki 2021 [8]	Japan	Small solitary HCC (< 5cm)	Age; sex; HBV; HCV; ICG-R15; Child-Pugh grade; tumor size; AFP; tumor number	132	66	66	8
Hokuto 2018 [9]	Japan	Extended to a depth of < 3 cm from the liver surface and measured < 5cm in diameter	Age; Gender; HBsAg; HCV-Ab; AFP; DCP; AST; ALT; albumin; operation time; tumor size; microvascular invasion	40	20	20	8
Ishii 2014 [10]	Japan	HCC patients undergoing hepatectomy	Age; gender; albumin; bilirubin; PT; size; Tumor number; Operation time; blood loss	88	44	44	7
Kaibori 2017 [11]	Japan	Curative resection; Solitary tumor ≤ 5cm; ICG-R15 ≤ 15%;	Age; HCV; HBV; bilirubin; ALT; albumin; PLT; ICG-R15; AFP; blood loss; operative time; size; MVI	710	355	355	8
Kwon 2022 [13]	Korea	Curative resection; Solitary tumor ≤ 5cm	Age; sex; HBV; HCV; Child-Pugh grade; PLT; total bilirubin; ICG-R15	341	224	117	7
Minagawa 2022 [12]	Japan	Solitary HCC (≤ 5cm)	Age; sex; HBV; HCV; total bilirubin; AST; ALT	134	67	67	8

*HCV* hepatitis C virus, *HBV* hepatitis B virus, *MVI* microvascular invasion, *PT* prothrombin time, *PLT* platelet count, *AFP* alpha-fetoprotein, *ALT* alanine-aminotransferase, *AST* aspartate-aminotransferase, *ICG-R15* Indocyanine Green Retention Test at 15 min, *DCP* Des-gamma-carboxy prothrombin, *MELD* model for end-stage liver disease, *AR* anatomic resection, *NAR* non-anatomic resection

### Basic characteristics of included studies and baseline characteristics of patients

#### Basic characteristics

The basic characteristics of included studies were recorded. These included the first author, year of publication, selection of subjects, and variables used for matching while performing propensity analysis, as shown in Table 1.

#### Baseline characteristics

Within the included literature, study subjects were classified by gender, liver function, hepatitis B and/or C infection, isolated nodules, microvascular infiltration, and perioperative morbidity contributing to within-study variations (see Table 2). Overall, in 8 studies, the percentages of female patients who underwent AR and NAR were 21.4% and 20.6%, respectively (*P* = 0.40; *I*^2^ = 4%). The vast majority of patients presented with Child-Pugh class A liver function (7 studies with 99.3% AR and 98.9% NAR; *P* = 0.72; *I*^2^ = 0%) and single nodules (8 studies with 95.0% AR and 96.7% NAR; *P* = 0.51, *I*^2^ = 0%); a lower percentage of patients exhibited combined microvascular invasion in 8 studies (AR, 24.0%, NAR, 26.2%; *P* = 0.98; *I*^2^ = 0%); 6 studies reported on patients with a history of hepatitis, with 46.5% and 43.3% of them undergoing AR and NAR, respectively, for hepatitis B testing; *P* = 0.79, *I*^2^ = 0%; 32.1% AR and 33.7% NAR for hepatitis C; *P* = 0.99, *I*^2^ = 0%; a total of 4 studies reported perioperative complications, of which 3 reported overall complications (AR, 20.0%, NAR, 18.1%; *P* = 0.41; *I*^2^ = 0%) and 2 reported major complications (AR, 5.7%, NAR, 4.6%; *P* = 0.34; *I*^2^ = 0%).

**Table 2:** Baseline characteristics of the whole sample considered for the meta-analysis

			AR		NAR				
		*N* of studies with data	*N* (%)	*N* total	*N* (%)	*N* total	RR (95%CI)	*P*	*I*^2^
Sex	Female	8	211 (21.4%)	984	181 (20.6%)	877	1.05 (0.88, 1.26)	0.40	4%
Liver function	Child A	7	755 (99.3%)	760	752 (98.9%)	760	1.00 (0.99, 1.02)	0.72	0%
Viral hepatitis status	HBV	6	410 (46.5%)	881	335 (43.3%)	774	1.01 (0.96, 1.06)	0.79	0%
	HCV	6	283 (32.1%)	881	261 (33.7%)	774	1.04 (0.91, 1.19)	0.99	0%
Number of nodules	Single	8	935 (95.0%)	984	848 (96.7%)	877	0.99 (0.98, 1.01)	0.51	0%
Microvascular invasion	Presence	8	236 (24.0%)	984	230 (26.2%)	877	0.99 (0.85, 1.14)	0.98	0%
Morbidity	Overall complications	3	88 (20.0%)	441	80 (18.1%)	441	1.10 (0.84, 1.44)	0.41	0%
	Major complications	2	5 (5.7%)	87	4 (4.6%)	87	1.25 (0.35, 4.51)	0.34	0%

Categorical variables are represented as number (%). Risk ratio(R) and 95% confidence interval (CI)

### Results of meta-analysis

#### Disease-free survival

**Fig. 2:**
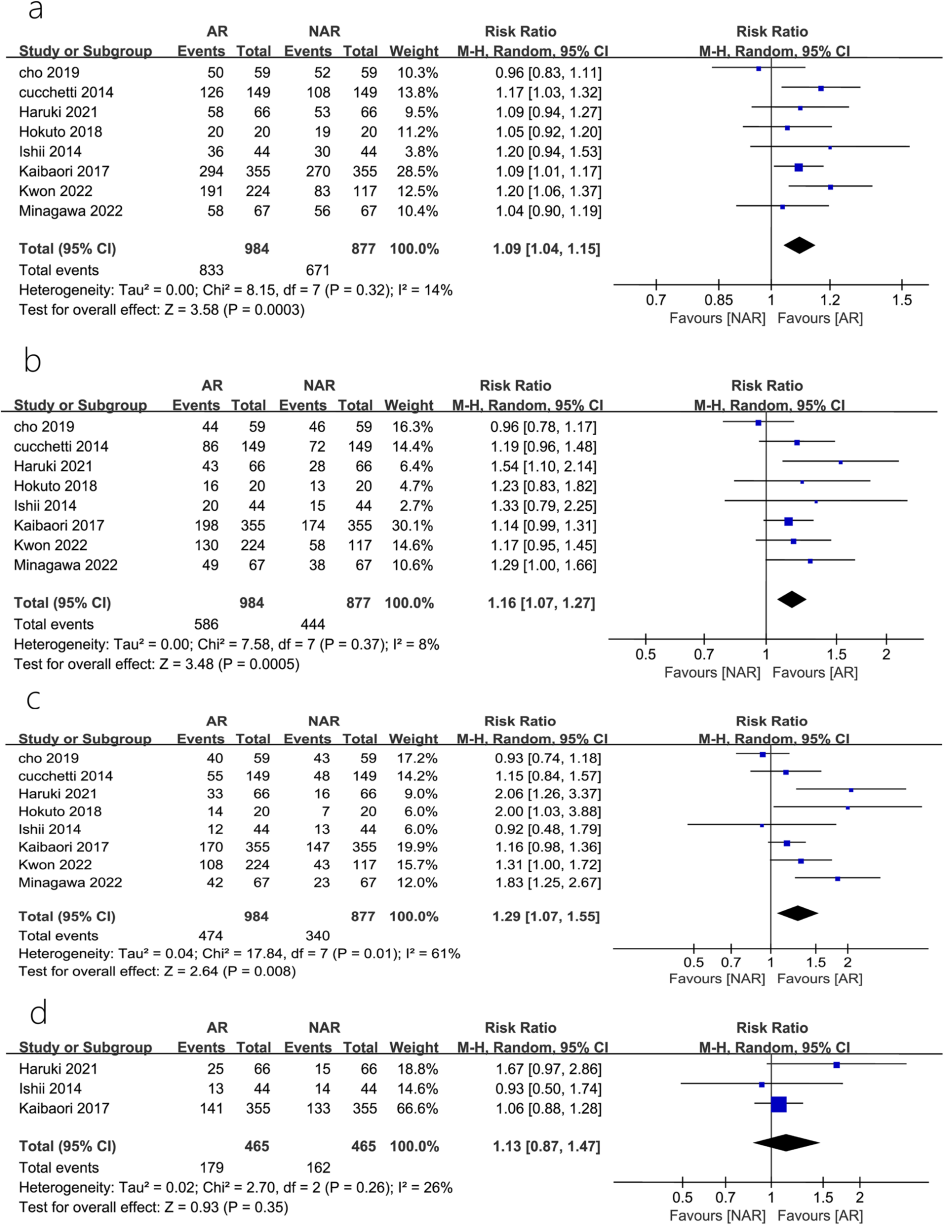
Forest plots of the effect of AR vs. NAR on **a** 1-year disease-free survival, **b** 3-year disease-free survival, **c** 5-year dise**a**se-free survival, and **d** 7-year disease-free survival. Risk ratios (RR) shown with 95% confidence intervals

**Fig. 3:**
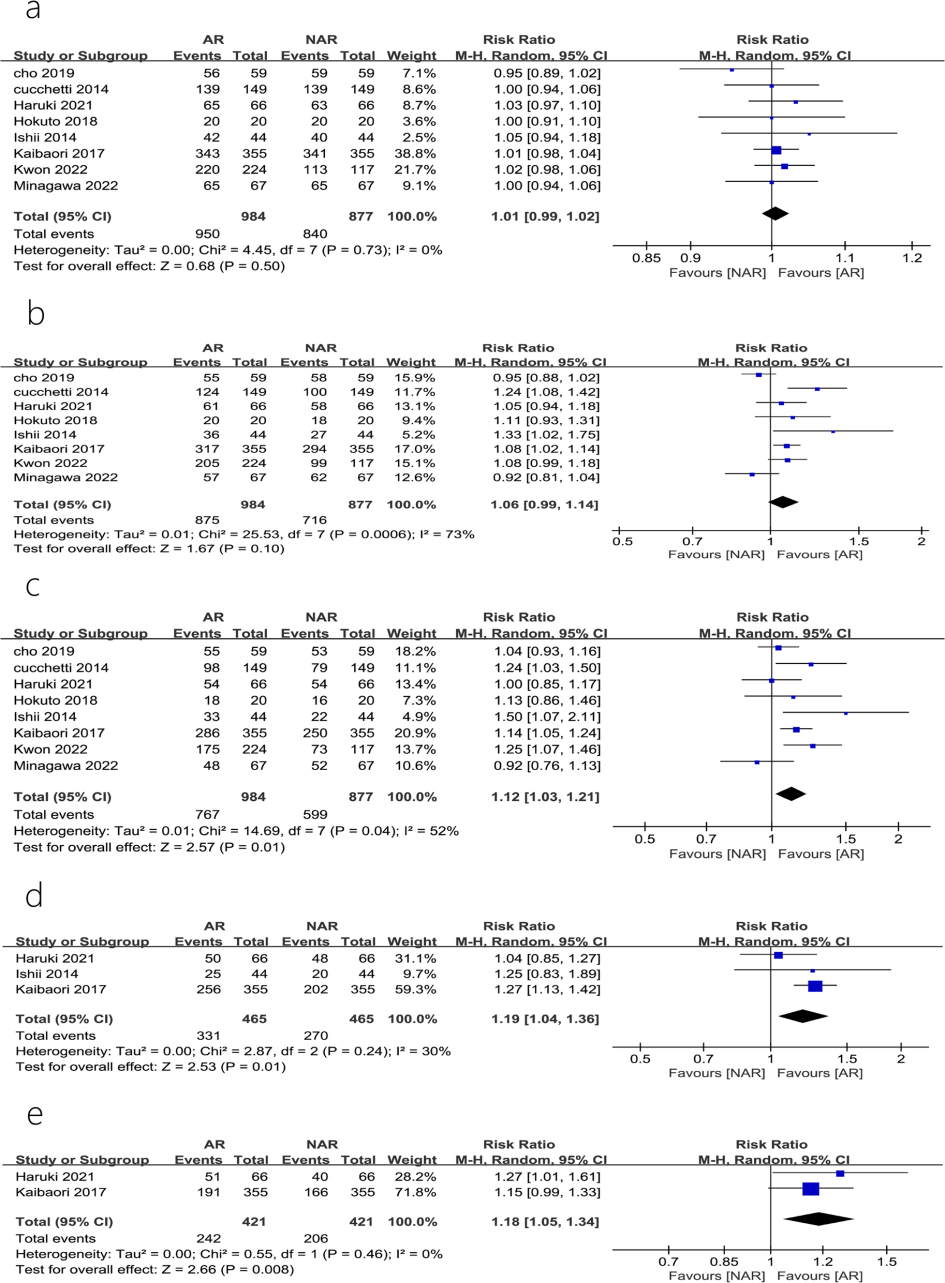
Forest plots of the effect of AR vs. NAR on **a** 1-year overall survival, **b** 3-year overall survival, **c** 5-year overall survival, **d** 7-year overall survival, and **e** 10-year overall survival. Risk ratios (RR) are shown with 95% confidence intervals

A total of 8 studies [7–14] reported 1-year, 3-year, and 5-year DFS; of these, 3 studies [8, 10, 11] reported a 7-year DFS. The heterogeneity tests performed in studies reporting 5-year DFS showed that there was heterogeneity between studies (*P* = 0.01, *I*^2^ = 61%). A sensitivity analysis, after omitting one study at the time, did not change the risk estimate or the level of significance for the outcome measures. The publication bias was evaluated by constructing a funnel plot, and the symmetry of the funnel plot suggested the absence of any publication bias (Fig. 2a). Meta-analysis using REM showed that AR significantly improved patients’ DFS postoperatively at 1, 3, and 5 years, compared to that observed with NAR (RR = 1.09, 95% CI = 1.04–1.15, *P* = 0.0003; RR = 1.16, 95% CI = 1.07–1.27, *P* = 0.0005; RR = 1.29, 95% CI = 1.07–1.55, *P* = 0.008) (Fig. 3a–c). However, the differences in DFS at 7 years after AR versus those after NAR were not statistically significant (RR = 1.13, 95% CI = 0.87–1.47, *P* = 0.35) (Fig. 3d).

#### Overall survival

**Fig. 4:**
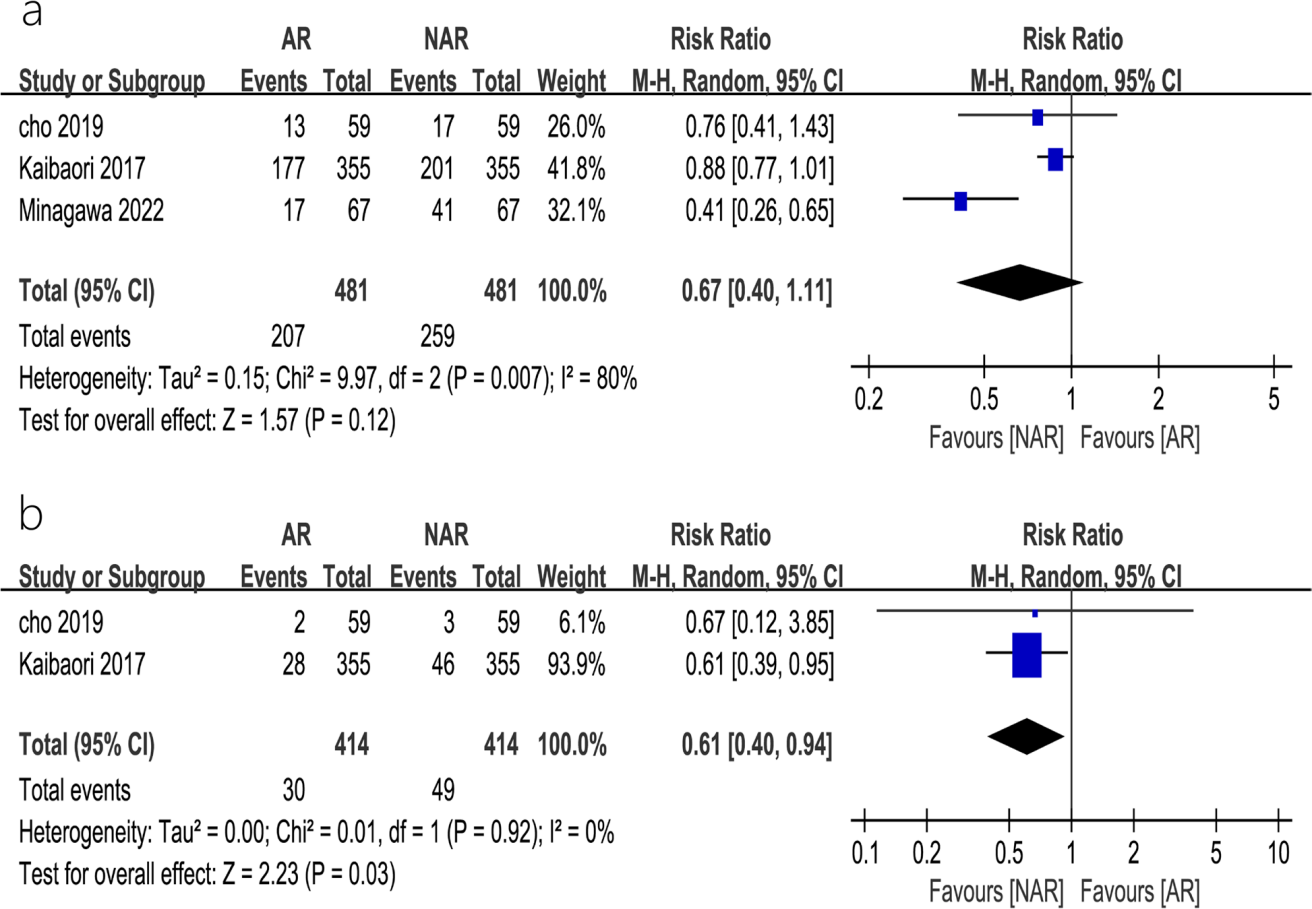
Forest plots of the effect of AR vs. NAR on **a** intrahepatic recurrence rate and **b** extrahepatic metastasis rate

A total of 8 studies [7–14] reported the OS at year 1, 3 and 5; of these, 3 studies [8, 10, 11] reported a 7-year OS and 2 studies reported [8, 11] a 10-year OS. The 3-year and 5-year OS heterogeneity test showed that there was heterogeneity among studies (*P* = 0.0006, *I*^2^ = 73%; *P* = 0.04, *I*^2^ = 52%). A sensitivity analysis, after omitting one study at the time, did not change the results. The related funnel plots for publication bias are shown in Fig. 2b, c. The results of a meta-analysis performed using REM showed that the differences in OS at 1 and 3 years after AR and NAR were not statistically significant (RR = 1.01, 95% CI = 0.99–1.02, *P* = 0.50; RR = 1.06, 95% CI = 0.99–1.14, *P* = 0.10) (Fig. 4a, b). However, AR was superior to NAR in terms of long-term OS (5, 7, and 10 years) (RR = 1.12, 95% CI = 1.03–1.21, *P* = 0.01; RR = 1.19, 95% CI = 1.04–1.36, *P* = 0.01; RR = 1.18, 95% CI = 1.05–1.34, *P* = 0.008) (Fig. 4c–e).

#### Intrahepatic and extrahepatic metastasis rates

**Fig. 5:**
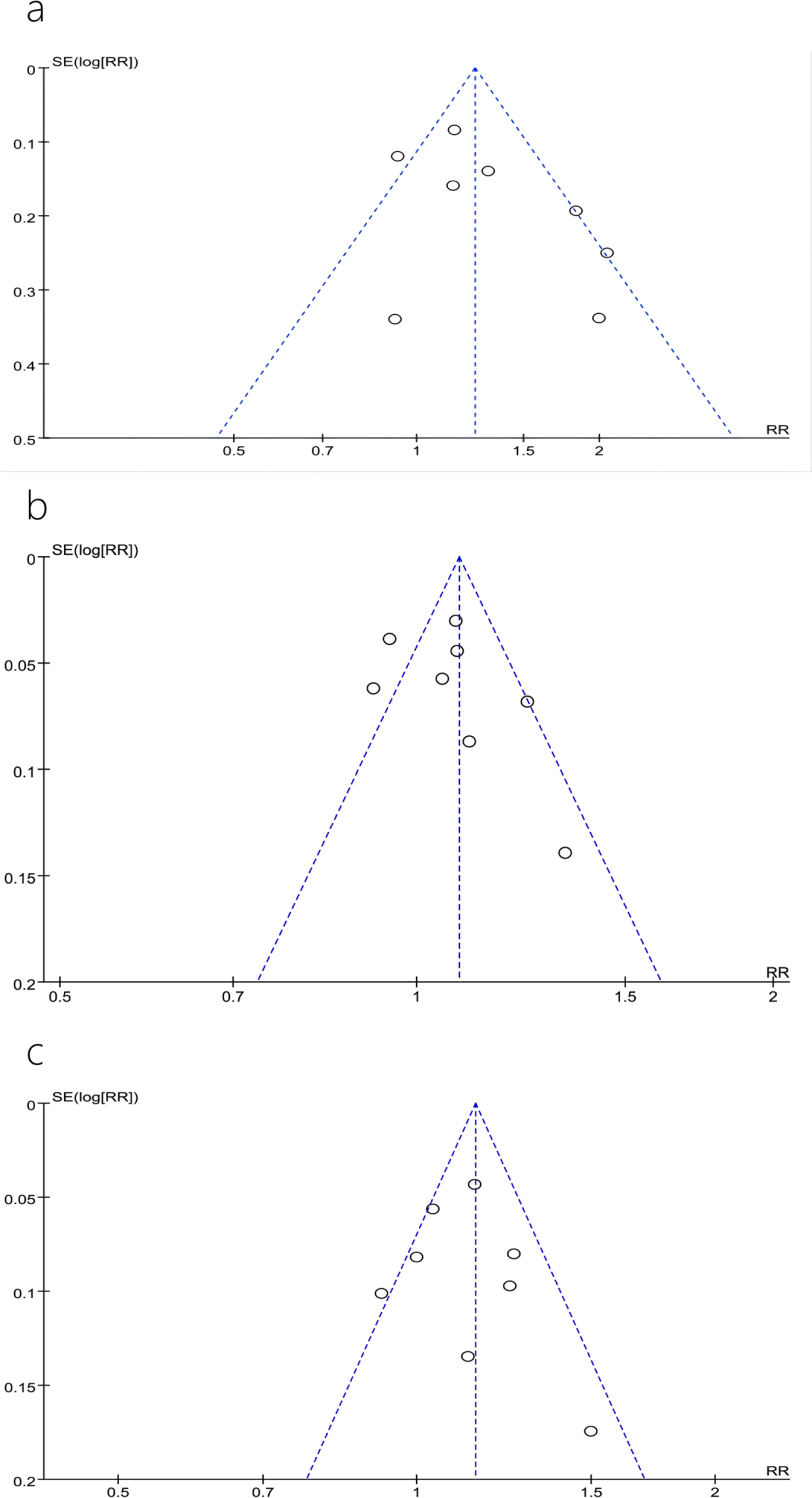
Funnel plots of the effect of AR vs. NAR on **a** 5-year disease-free survival, **b** 3-year overall survival, and **c** 5-year overall survival

A total of 3 studies [11, 12, 14] reported intrahepatic recurrence rates and 2 studies reported [11, 14] extrahepatic metastasis rates, and the results of the heterogeneity test, performed using REM, showed that there was heterogeneity among studies (*P* = 0.007, *I*^2^ = 80%). Sensitivity analysis did not significantly change the results. The results of the meta-analysis showed that the difference in intrahepatic recurrence rates after AR versus that observed after NAR was not statistically significant (RR = 0.67, 95% CI = 0.40–1.11, *P* = 0.12) (Fig. 5a), but the incidence of extrahepatic metastasis after AR was significantly lower than that observed after NAR (RR = 0.61, 95% CI = 0.40–0.94, *P* = 0.03) (Fig. 5b).

## Discussion

Liver surgery techniques have been developed over several stages, via techniques such as wedge resection, regular lobectomy, irregular partial resection, and anatomical segmental hepatectomy [18]. In the mid-1950s, Goldsmith and Woodbume [19] emphasized that lobectomy should be performed strictly in accordance with the internal anatomy of the liver, thus introducing the concept of a regular lobectomy. In 1985, Makuuchi et al. [20] used AR for the first time for the treatment of HCC, which resulted in the currently accepted concept of AR. From the anatomical point of view alone, AR has many advantages over NAR. First, AR can simultaneously remove tumors and intrahepatic disseminated micrometastases [21]. In addition, several previous studies have shown that HCC patients exhibit lower rates of recurrent metastases, higher survival rates, and better prognoses after AR [7, 22, 23].

However, in practical clinical work, the application of AR is limited by several factors. For perioperative safety reasons, surgeons prefer AR for patients exhibiting good liver function (Child A), small tumor size (e.g., tumor diameter ≤ 5 cm), and no cirrhosis, leading to an unavoidable selection bias [22]. Therefore, there is a long-standing debate on whether the choice of AR or NAR is more beneficial and results in a better long-term prognosis for HCC patients. On one hand, separate case-control studies addressing these issues have led to large discrepancies in results due to the use of different criteria for patient selection (because of differences in clinical characteristics, such as basic preoperative characteristics, liver function classification, tumor number, and vascular invasion). On the other hand, though some meta-analyses have addressed these issues, there is a natural inherent cohort bias that affects the conclusiveness of the results [24, 25]. This suggests that there is an urgent need for a more scientific approach for the analysis of the above issues. The concept of PSM was first proposed by Rosenbaum and Rubin in 1983, and the method reduces bias and ensures that baseline information is balanced between groups [16]. In the absence of RCTs, PSM can reasonably match two groups of patients to achieve an approximation of a randomized controlled clinical study, thus ensuring balanced comparability. Therefore, we used PSM to reduce the differences in variables between groups, and again compared the OS, DFS, and recurrent metastasis rates of HCC patients with a tumor diameter ≤ 5 cm who underwent AR and NAR. Our study reduced the heterogeneity of the included studies to some extent, and analyzed more distantly related indicators such as 7-year DFS and OS; thus, our study is more convincing than previous meta-analyses.

Patients with HCC have a high mortality rate and poor prognosis, with an overall 5-year survival rate of only 18% [26] and a cumulative recurrence rate of 70-80% even 5 years after undergoing radical surgery [27]. Studies have shown that microvascular invasion (MVI) is an independent risk factor affecting the prognosis of HCC patients [28]. Before performing liver resection in HCC patients with combined MVI, tumor cells may already be present in the remnant liver and exhibit microscopic portal infiltration and the formation of satellite foci, which in turn might allow early tumor recurrence and metastasis [29, 30]. In this study, we found that the DFS was higher in the AR group than in the NAR group at 1, 3, and 5 years after surgery, probably because AR was based on the systematic distribution of the portal vein, which reduced the spread of tumor cells in the portal system and decreased the possibility of tumor metastasis. In addition, postoperative extrahepatic metastases usually occur later and are more lethal than intrahepatic recurrence for HCC patients [31–33]. In this study, we found that the rate of extrahepatic metastasis was higher in NAR than in AR, which could be attributable to the fact that there were no significant differences in the DFS for the procedures at 7 years after surgery.

It was found that the probability of intrahepatic metastasis in the short term (within 2 years) after liver cancer surgery was about 0.68, while extrahepatic metastasis was predominant over the long term [34]. The cumulative survival rate associated with intrahepatic recurrence was significantly higher than that associated with extrahepatic metastasis [35]. In this study, we found that there was no significant difference in the rate of intrahepatic recurrence after AR and NAR, which may explain the lack of significant difference in the short-term OS (1, 3 year) between the two groups. The long-term OS (5, 7 and 10 years) was significantly higher for the AR group than the NAR group, which may be attributed to the higher rate of extrahepatic metastasis associated with NAR, compared to AR. Therefore, this study suggests that in HCC patients with tumor diameters ≤ 5 cm, AR can effectively reduce the rate of postoperative extrahepatic metastasis and thus improve the postoperative long-term OS.

Nevertheless, there are some limitations to this study. First, the selection criteria of patients were varied; important indicators such as the ICG15 level, the occurrence of MVI, and cutting-edge condition did not help us develop uniform criteria. Some previous studies [6, 36] have shown that the cutting-edge condition did not influence the recurrence rates after hepatectomy for HCC. Most intrahepatic recurrences were considered to arise from intrahepatic metastasis by venous dissemination, which a wide resection margin could not prevent. However, recent studies [37–41] suggest that HCC patients with narrow resection margin were associated with a higher tumor recurrence rate and a shorter DFS. A multicenter retrospective study [42] concluded that AR with a negative 0-mm surgical margin may be acceptable in patients with a single hepatocellular carcinoma. NAR with a negative 0-mm margin was associated with a less favorable survival outcome. For better oncologic outcomes, surgeons should endeavor in keeping the surgical resection margin widths ≥1 cm during NAR. Furthermore, there are differences in prognostic outcomes due to the different etiologies of patients in the East and West. Finally, even if we use PSM as a scientific method, an intergroup error was still observed between the included studies. Therefore, a multi-center, randomized controlled study needs to be performed, to further investigate the impact of these two procedures on the long-term prognosis of HCC patients.

In conclusion, AR is superior to NAR in terms of both the short-term DFS and long-term OS of HCC patients with tumor diameters ≤ 5 cm. Therefore, whenever feasible, AR should be the preferred surgical approach for HCC patients with tumor diameters ≤ 5 cm.

## Data Availability

All data generated or analyzed during this study are included in this published article and its supplementary information files.
